# Extraction and visualization of orientation data from virtual geologic surfaces with MATLAB®

**DOI:** 10.1016/j.mex.2019.10.033

**Published:** 2019-11-02

**Authors:** Avery J. Welker, John P. Hogan, Andreas Eckert

**Affiliations:** Department of Geosciences and Geological and Petroleum Engineering, Missouri University of Science and Technology, Rolla, MO, USA

**Keywords:** Using MATLAB® to extract and visualize planar geologic attitudes from point-based data, Planar data visualization, Structural geology, Stereographical analysis, Polar tangent diagram

## Abstract

High-resolution visualization of surfaces of geologic interest, at a multitude of scales, using 3D point cloud technologies provides an opportunity to analyze spatial relationships of surfaces using orientation data. We present a MATLAB® script that produces planar geologic attitude data (e.g., strike, dip, and dip-direction data) from 3D datasets (e.g., point clouds, 3D scanning). The method utilizes Cartesian coordinates of triangular planar surfaces and converts them into matrices of conventional geologic attitude data. Spatial relationships among data points can be investigated, using polar tangent diagrams, stereographic analysis, or geologic curvature analysis. We utilize this script to create “synthetic” graphical plots (e.g., stereograms, tangent diagrams) from geomechanically realistic, virtual, folded surfaces produced by dynamic modeling. Synthetic graphical diagrams are of considerable usefulness in interpreting graphical plots (e.g., stereograms) of attitude data from natural folded rock surfaces, particularly in locations with poor exposure.

• This script outputs attitude data (strike, dip, and dip direction) in a spreadsheet and as a text file for use in other visualization software.
• A tangent diagram is created and displayed in this script for rapid visualization and fold shape assessment.
• The MATLAB script is readily modified to accept multiple data formats for input (e.g., MATLAB variables, *.csv files, etc.) and output (e.g., *.csv files, *.txt files, etc.).

## Graphical abstract

**Figure:**
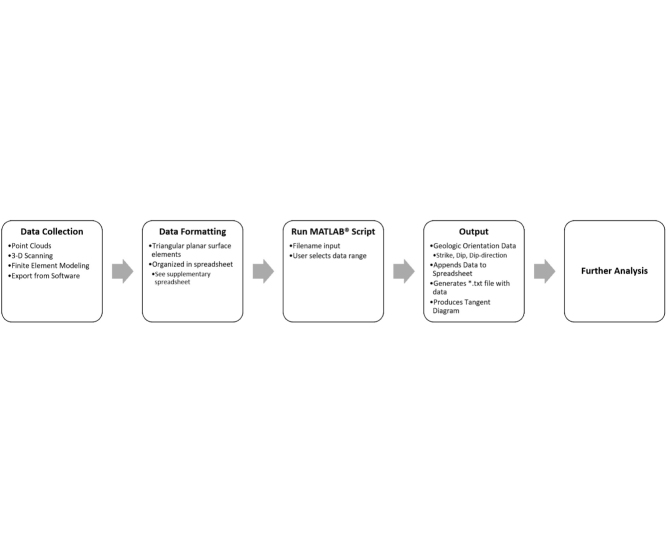

## Specification Table

**Table:** 

Subject Area:	*Earth and Planetary Sciences*
More specific subject area:	*Extracting attitude data from virtual surfaces of geologic structures*
Method name:	*Using MATLAB® to extract and visualize planar geologic attitudes from point-based data*
Name and reference of original method:	*The Three-Point Method to Determine Strike and Dip from Hasbargen, L.E., 2012, A test of the three-point vector method to determine strike and dip utilizing digital aerial imagery and topography, in Whitmeyer, S.J., Bailey, J.E., De Paor, D.G., and Ornduff, T., eds., Google Earth and Virtual Visualizations in Geoscience Education and Research: Geological Society of America Special Paper 492, p. 199-208, doi:10.1130/2012.2492(14).* *Tangent Diagram on a Spreadsheet from* [8]*. 3-D Structural Geology – A Practical Guide to Quantitative Surface and Subsurface Map Interpretation. Springer-Verlag.*
Resource availability:	MATLAB, Microsoft Excel

## Method details

Graphical plots such as stereograms and tangent diagrams are common visual tools, which geologists use to analyze spatial relationships among planar and linear elements in rocks ([1], p. 366 and 368). These diagrams are an important tool in the characterization of folds, as they can help classify the shape of a fold (e.g., cylindrical or non-cylindrical), and of the overall orientation of the fold in space (i.e., strike and dip of the axial surface, plunge and trend of the hinge line). Traditionally, characterization of strain in rocks begins with the collection of orientation data (e.g., strike, dip, and dip-direction) of planar surfaces and linear elements using a geologic compass. Subsequent analysis of this data using graphical plots can lead to equivocal interpretations, particularly for sparse data sets, for example, from folded rocks that are poorly exposed. To reduce the ambiguity in interpreting graphical plots of natural folds, Welker et al. [2] utilized the approach of extracting orientation data from high-resolution 3D virtual folds to create “synthetic” stereograms and tangent diagrams. These virtual folds are the product of geomechanically realistic dynamic modeling (see [3]) and are appropriate complimentary surfaces to use for direct comparison with natural folds. With this approach, Welker et al. [2] demonstrated that orientation data for natural folds, which define small circles on stereograms, are compatible with geomechanically realistic fold shapes known as “periclines” (i.e., doubly plunging antiforms and synforms) (see [4] p. 262) and are not *a priori* indicative of a conical shape for folds.

We present the method used by Welker et al. [2] to extract high spatial resolution orientation data (i.e., strike and dip) from virtual 3D surfaces of geologic structures and create synthetic graphical plots (i.e., polar tangent diagrams). In addition, the orientation data can be used by other, existing software packages (e.g., Stereonet 10, see [5] and [6], Move™) for creation of additional diagrams (e.g., stereonets, geologic curvature analysis). This method utilizes a MATLAB script employing equations of the three-point vector method [7] to generate geologic attitude data from Cartesian coordinates of triangular planar surfaces and create a tangent diagram ([8], p. 47). 3D geologic surfaces are commonly characterized as point cloud data (e.g., LiDAR). The point cloud data is then discretized into high-resolution triangular surfaces using commonly available geologic modeling packages such as Petrel® and GOCAD®, or by using finite element pre-processors such as HyperMesh®. The script accepts Cartesian coordinates of triangular planar surfaces from a comma separated variable ASCII text file, *.csv, or Microsoft Excel® spreadsheet. The script then generates an ASCII text file (*.txt) and a new spreadsheet containing strike, dip, and dip direction (right-hand rule) data. Modification of inputs and outputs in the script are easily performed to fit the data format needs of an individual user. Descriptions for each variable found in the script are shown in Table 1. This method can be readily adapted to produce planar geologic attitude data (e.g., strike, dip, and dip-direction data) from other 3D datasets with associated Cartesian coordinates such as seismic surveys, point clouds, and 3D scanning.

**Table 1:** Variable list and descriptions.

Variable	Explanation
prompt	Text to be displayed when MATLAB prompts for input of a filename
filename	Stores the name of the file as a string as input in MATLAB
data	Cumulative matrix of all user selected Cartesian coordinate data
numRows	Counts the number of rows in the 'data' matrix for use in creating the strike vector
XYZ1	Matrix of first finite element coordinate split into columns
XYZ2	Matrix of second finite element coordinate split into columns
XYZ3	Matrix of third finite element coordinate split into columns
vector1	Normal vector of 'XYZ2' to 'XYZ1'
vector2	Normal vector of 'XYZ2' to 'XYZ3'
normalV	Cross product of 'vector2' and 'vector1'
strikeV	Vector holding cross product of 'normalV' and the pole to an arbitrary horizontal plane
h	For loop counting variable
bearing	Matrix of pre-azimuthal corrected strike data
i	For loop counting variable
azimuth	Matrix of azimuthal strike data
j	For loop counting variable
dip	Matrix of dip data
k	For loop counting variable
dipDirection	Matrix of dip direction data
q	For loop counting variable
CombinedResults	Three-column matrix containing strike, dip, and dip direction data from 'azimuth,' 'dip,' and 'dipDirection' respectively
TangentPlotMatrix	Matrix combining dip and dip direction data from 'dip' and 'dipDirection' respectively
transformedData	Transformation of 'TangentPlotMatrix' from degrees to radians
TanDipRad	Matrix containing the tangent of dip data in radians
DipDirectionRad	Matrix containing dip direction data in radians
pax	Polar axis object
thetaLabels	Matrix of labels for theta axis of tangent diagram in degrees

3D characterization of geologic surfaces, above and below ground, from hand-sample to mountains, is becoming increasingly more important in modern-day and future spatial geoscientific data analysis (see Applications below). The method presented here accounts for the emerging importance of digital outcrop models (see [9]) created by these studies, and provides a tool/interface that is readily usable as is, or can be modified based on the MATLAB code provided.

**Fig. 1:**
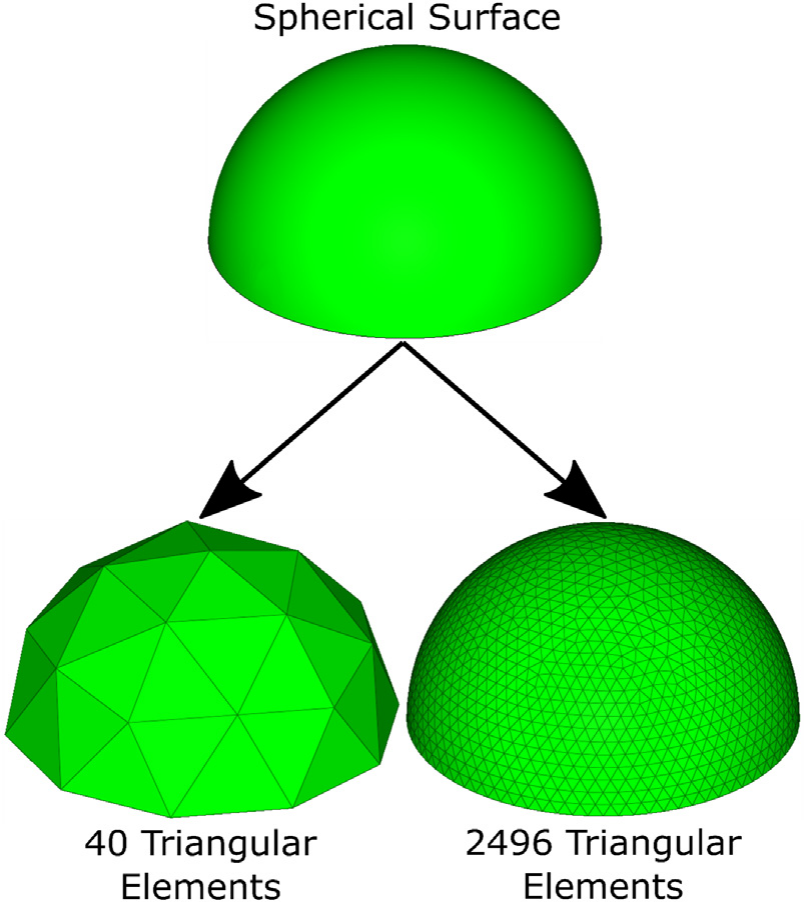
Example of a spherical surface discretized into triangular surface elements at high and low resolutions. A higher data resolution (bottom right) results in a more accurate representation of the spherical surface than a lower resolution (bottom left).

If the resolution of the point cloud data, as may be obtained by LiDAR, is not sufficient to provide an accurate representation of the surface geometry, the discretized (i.e., triangulated surface) geometry is prone to significant uncertainties and errors (Fig. 1).

## Procedure

### Surface data

**Fig. 2:**
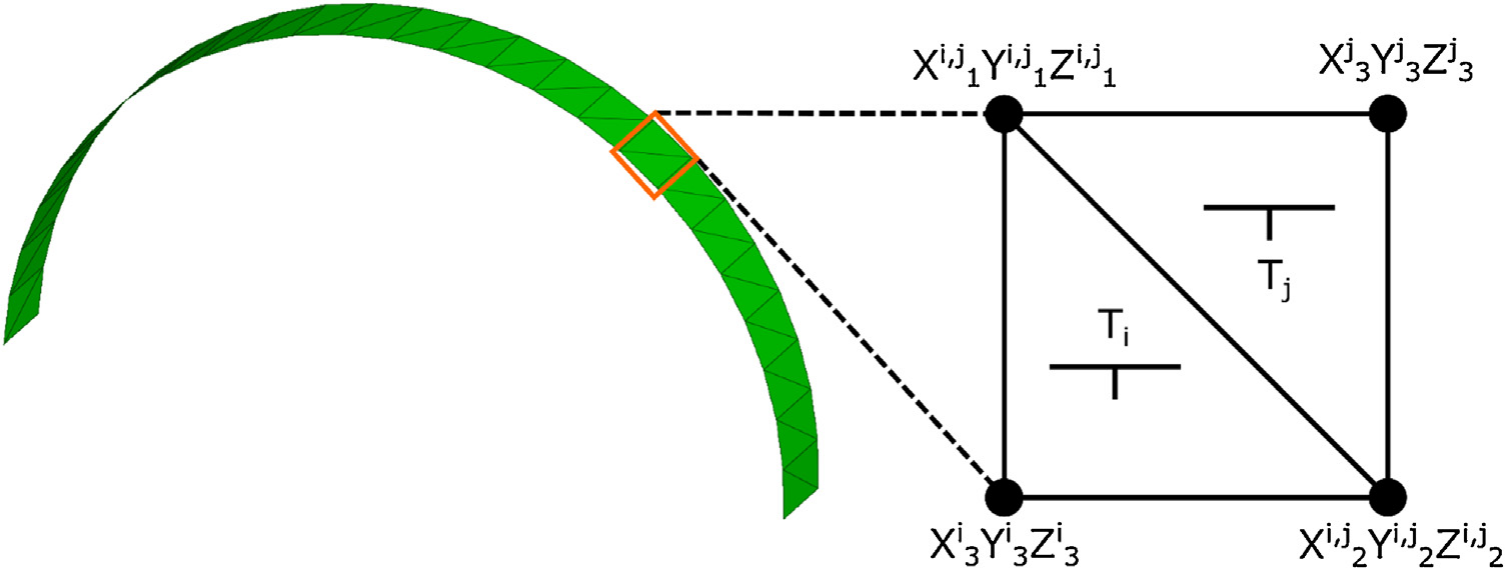
Example of a folded surface, S, discretized into triangular surface elements (T_i_ and T_j_). Coordinate point superscript identifiers correspond to a single row of data points in Fig. 2. Subscripts to coordinate points correspond to the Point 1, 2, and 3 labels in Fig. 2.

**Fig. 3:**
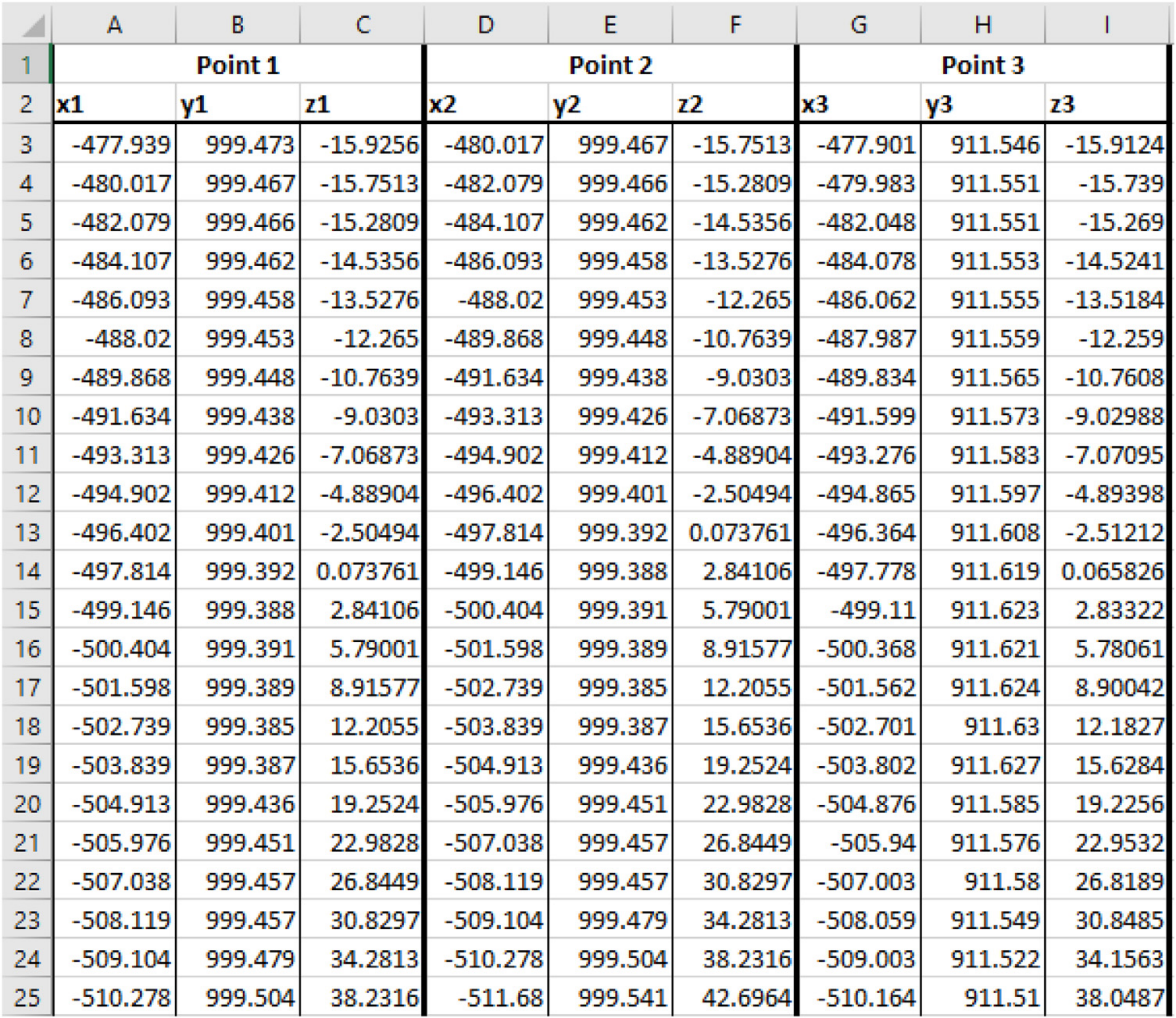
Example spreadsheet formatting.

The following MATLAB® script processes a triangulated surface, S (e.g., as obtained from a geological surface from LiDAR point clouds, or digitally from geological or numerical models), based on the Cartesian coordinates of its individual triangulated surfaces (T_i_). The individual coordinates of the three points defining T_i_ need to be in the following spreadsheet (i.e., .xls, .xlsx, .csv, .dat, etc.) format: For each T_i_ (organized in subsequent rows), 9 columns, giving the respective x, y, and z coordinates of the corners of the triangle, x^i^_1_, y^i^_1_, z^i^_1_, x^i^_2_, y^i^_2_, z^i^_2_, x^i^_3_, y^i^_3_, and z^i^_3_, are necessary (Fig. 2, Fig. 3, also see Cylindrical_Test_Data.xlsx in Supplemental material).

It needs to be noted that the accuracy of the following attitude calculations are critically dependent on triangulated mesh resolution of the surface being determined. It is the users’ responsibility to ensure that the triangular shapes approach equilateral triangles (i.e., aspect ratio of 1 and internal angles of 60° each) and that the resolution captures local variations in surface curvature (e.g., see [10]). If data resolution is not high enough, the authors suggest smoothing the dataset to improve results and avoid artefacts.

### Attitude calculation/main script

**Fig. 4:**
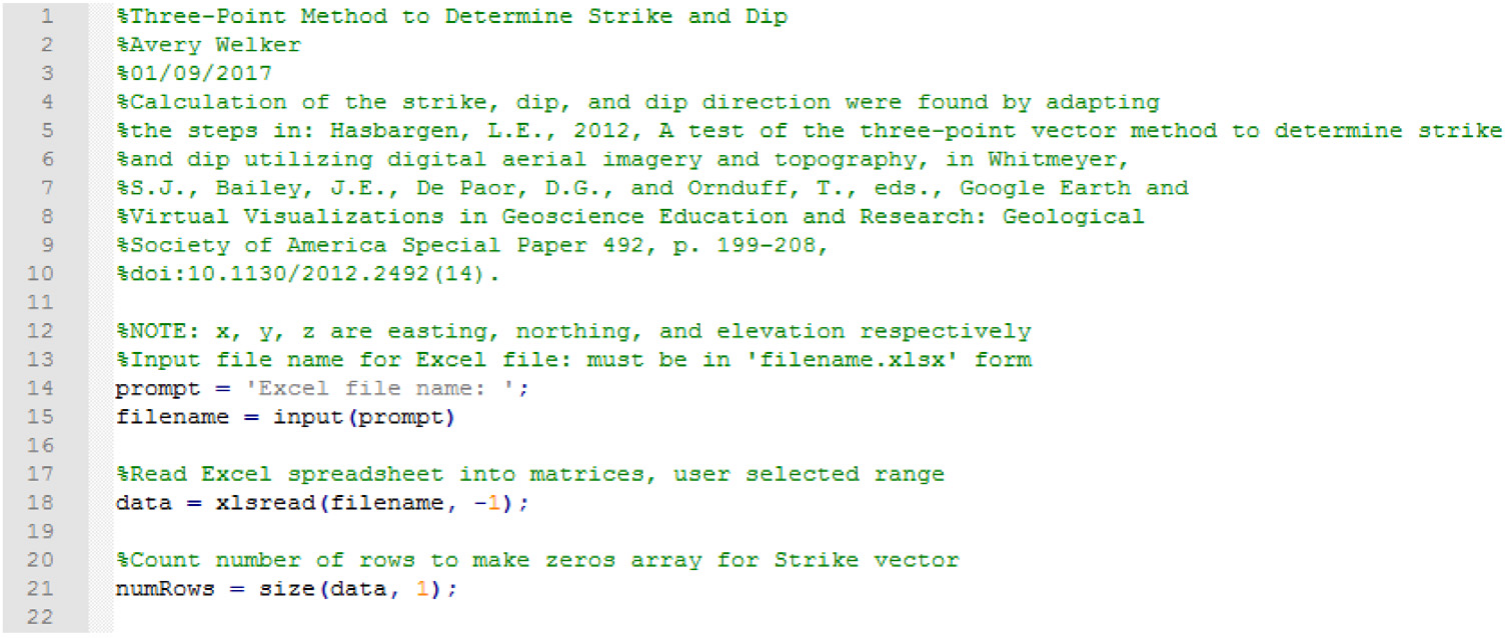
Script preamble, program input, and sizing of matrices.

Upon running the script, MATLAB prompts for the name of the spreadsheet, expecting ‘filename.extension’, by calling the MATLAB function “xlsread”. The spreadsheet then opens for user selection of data. Highlight all the data to be analyzed and select ‘OK’ on the MATLAB prompt. The dimensions of the matrix selected in the spreadsheet are used to pre-allocate matrices for computing efficiency when calculating bearing and strike (Fig. 4).

**Fig. 5:**
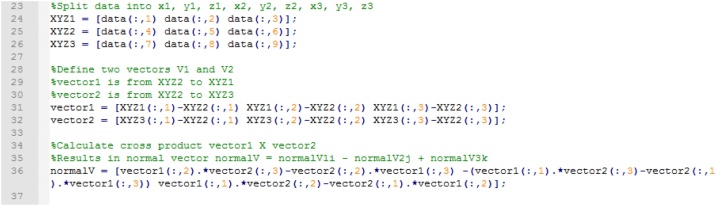
Splitting Cartesian coordinates into matrices, defining vectors of planes of interest, and calculating cross product of plane vectors.

Next, the matrix of Cartesian data is split into three matrices, corresponding to each individual point. The three points of each triangle are used to construct the two basis vectors of the plane embedding the triangle. The normal of the plane is obtained by computing the cross product of the two basis vectors (Fig. 5).

**Fig. 6:**
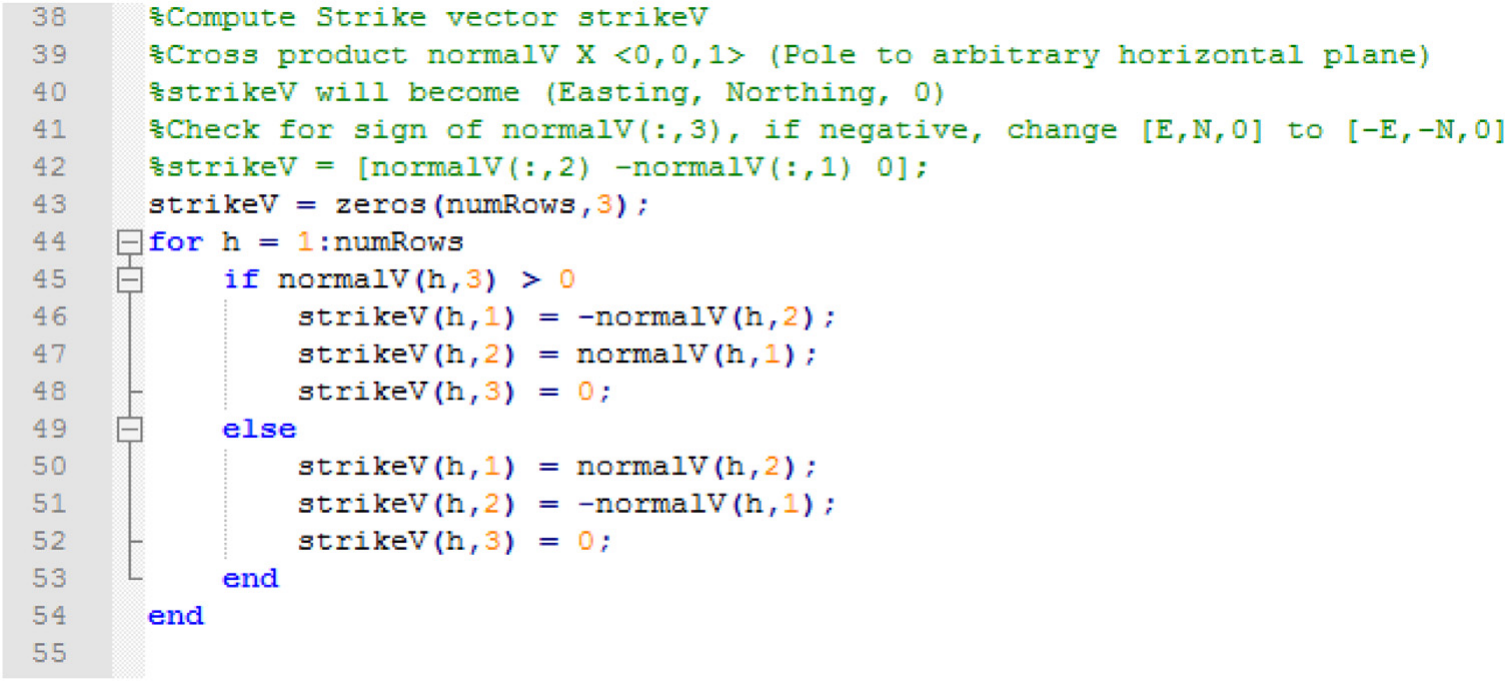
Computing strike line vector and checking/correcting polarity of vector.

A strike line is the intersection line between a horizontal plane and the one of interest. Its direction vector is calculated by computing the cross product of the normal vector of a horizontal plane [7]. The vector is created using one of the pre-allocated matrices, and a check is performed to ensure the polarity of the calculated strike vector is correct (Fig. 6) (see [7] for explanation).

**Fig. 7:**
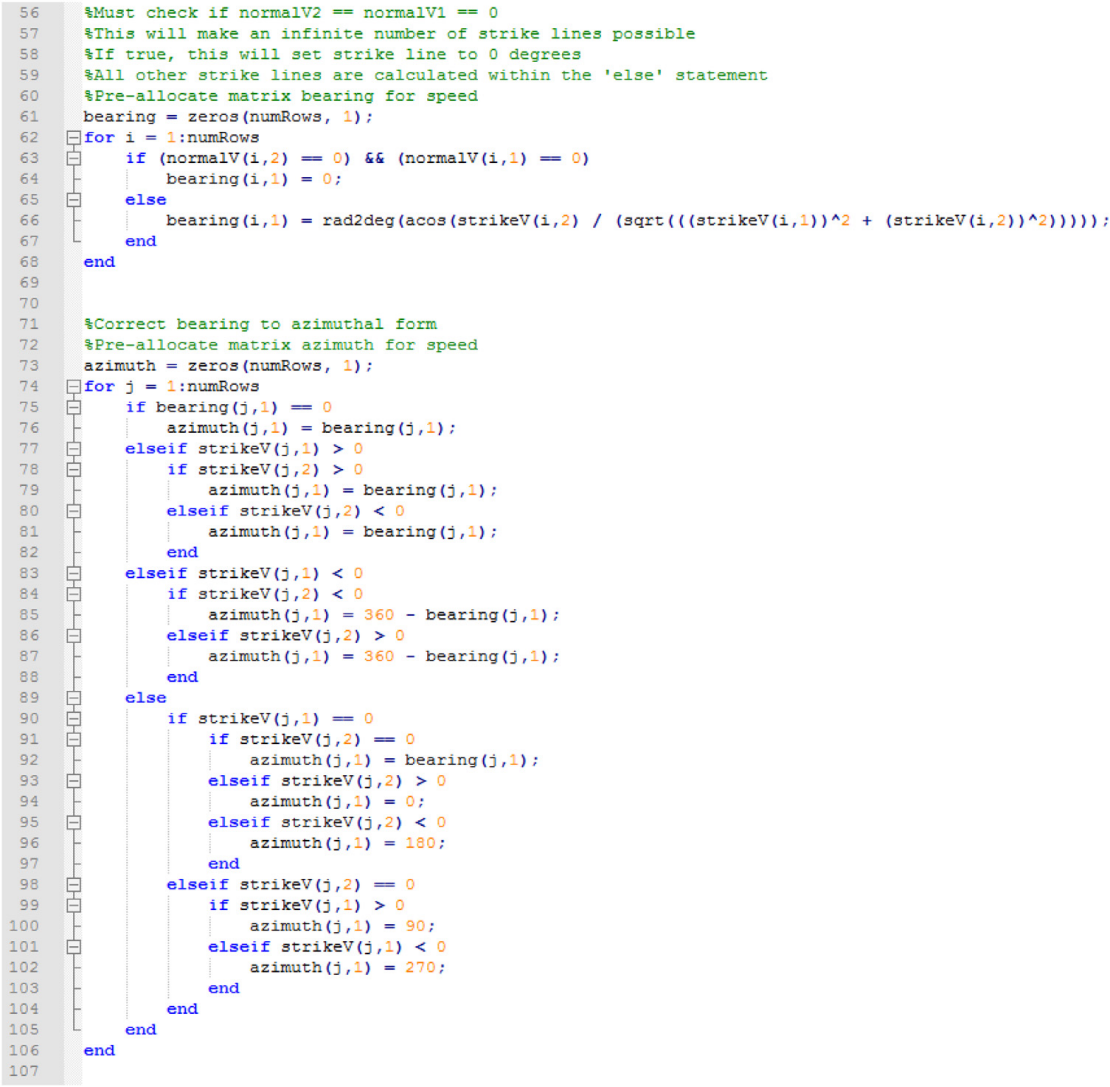
Assigning 000 strike value to horizontal planes and correcting bearing to azimuth.

The code then performs a check to see if the plane of interest is horizontal and assigns a strike value of 000 to it. If the plane is not horizontal, the bearing of the strike line is calculated. This calculation is then corrected to azimuth following the conversion table found in Hasbargen [7] (Fig. 7).

**Fig. 8:**
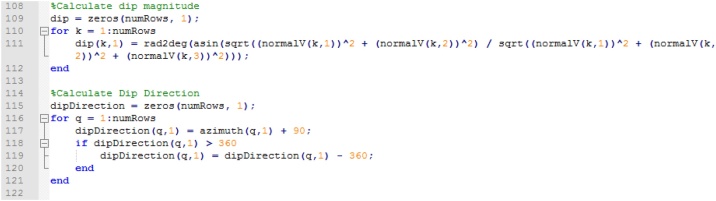
Calculating dip and dip direction (right hand rule).

The magnitude of the dip of each plane of interest is calculated with the components of the vector normal to the plane of interest (see [7]) and dip direction following the right-hand rule (Fig. 8).

**Fig. 9:**
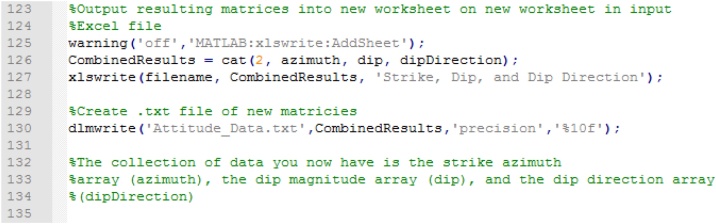
Organizing final matrices of results (strike, dip, and dip direction) and generating output of text file and additional spreadsheet to input Excel file.

Finally, the resulting matrices containing the strike (azimuth), dip, and dip direction are combined, and MATLAB appends a sheet containing this information to the input spreadsheet along with generating a separate text file (Fig. 9).

**Fig. 10:**
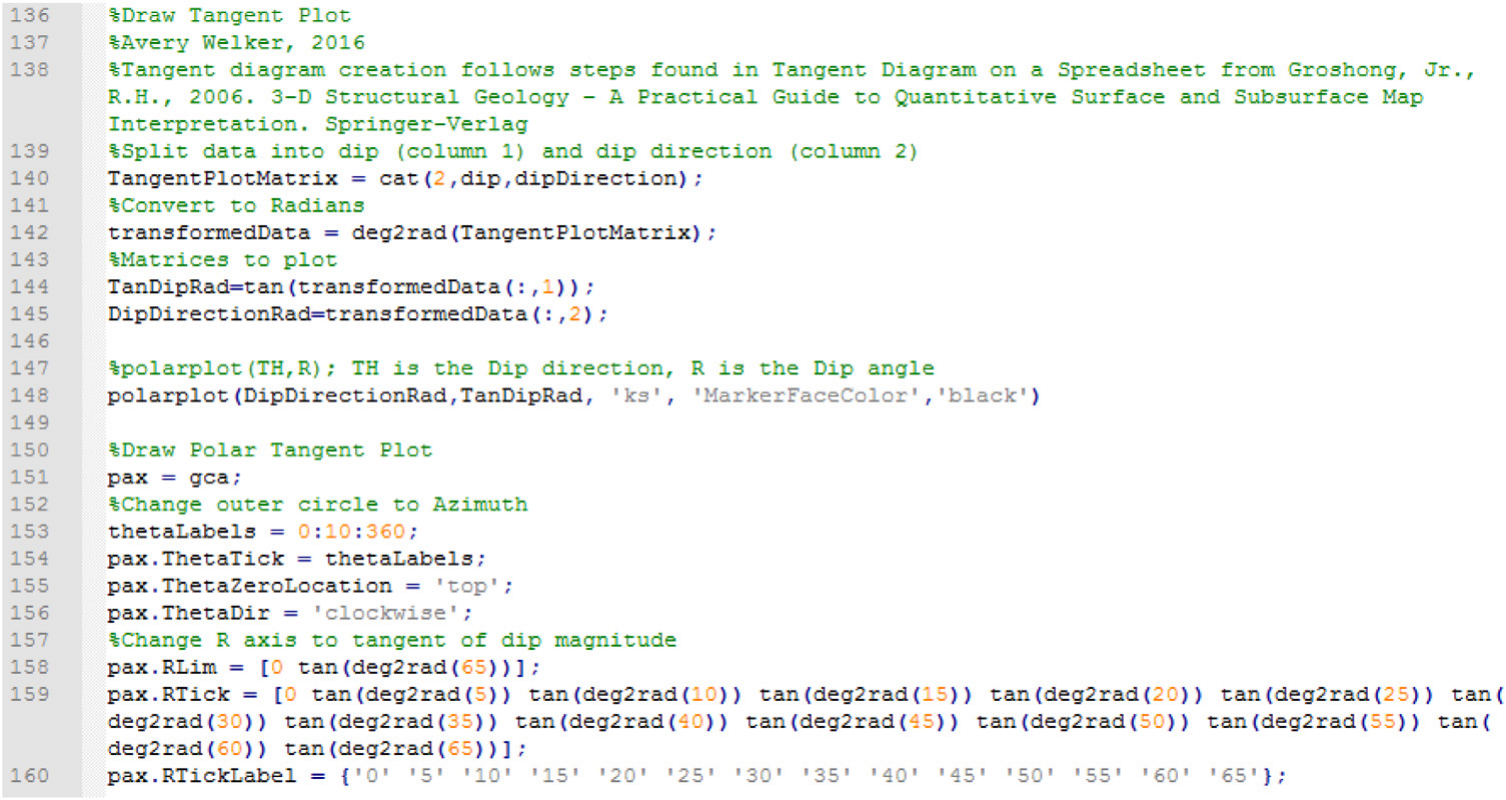
Generation of tangent diagram.

To draw the tangent diagram (following [8], p. 47), the code creates a matrix appending dip and dip direction together, transforms them to radians, and splits the matrix into two matrices, each containing radian values of dip and dip direction respectively (Fig. 10).

**Fig. 11:**
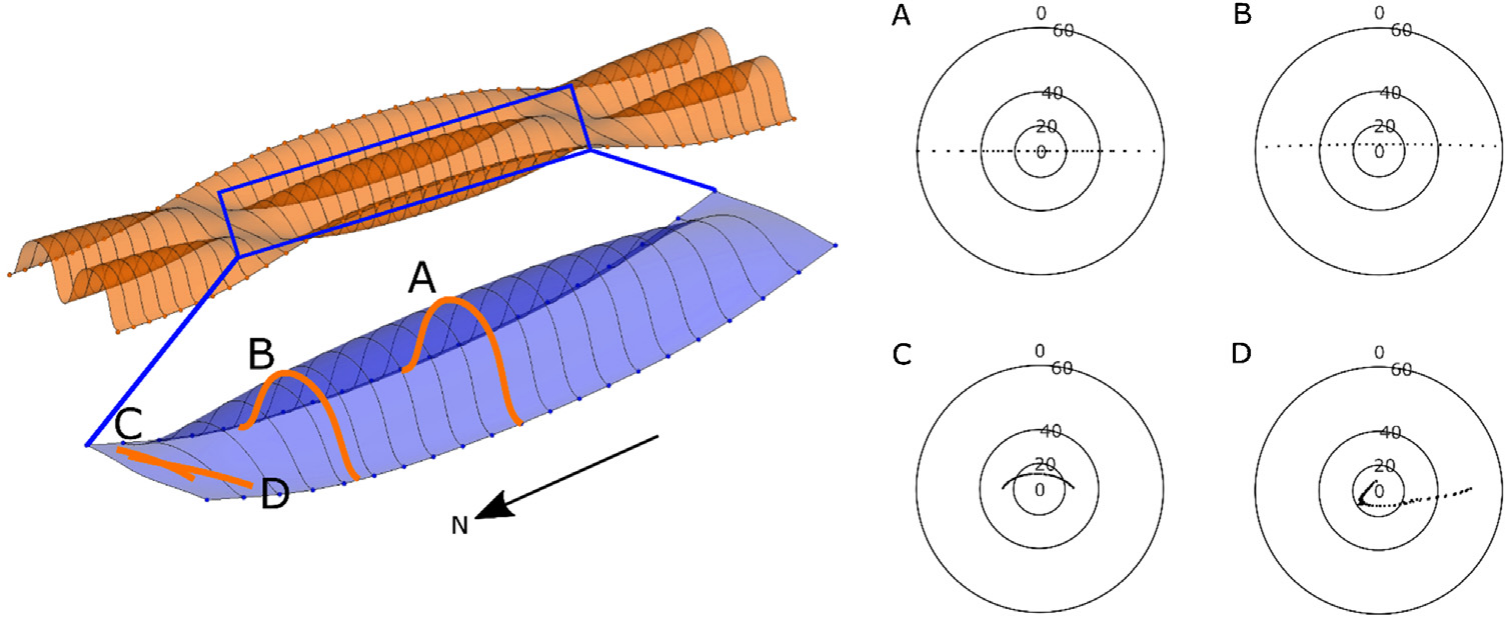
Example transects of surfaces in orange boxes. Each tangent diagram in A), B), C), and D) corresponds to the attitude data generated from virtual model of pericline (modified from [2]).

The tangent diagram is a polar plot featuring the dip as the radial axis, and the dip direction around the circumference ([8], p. 47). The script generates a tangent diagram with angular labels and is convenient to customize with MATLAB’s internal tools. Example input surfaces with generated tangent diagrams associated to each are illustrated in Fig. 11.

### Applications

Besides geometries obtained through numerical modeling approaches, virtual 3D geometries of geologic structures can be readily obtained: (a) in the field from drone based 3D scanning or LIDAR resulting in ultra-high resolution point cloud data sets; and (b) in analogue laboratory modeling experiments using laser scanning [11] or stereovision techniques [12]. In addition to the ultra-high spatial resolution of geometric data, if applied to virtual geometries developed at different time steps in a numerical or analogue model, the method presented can provide analysis of spatial-temporal change in geometric attitudes and geometric shape of a structure.

### Case study: the Whaleback pericline, Bear Valley Strip Mine, Pennsylvania

**Fig. 12:**
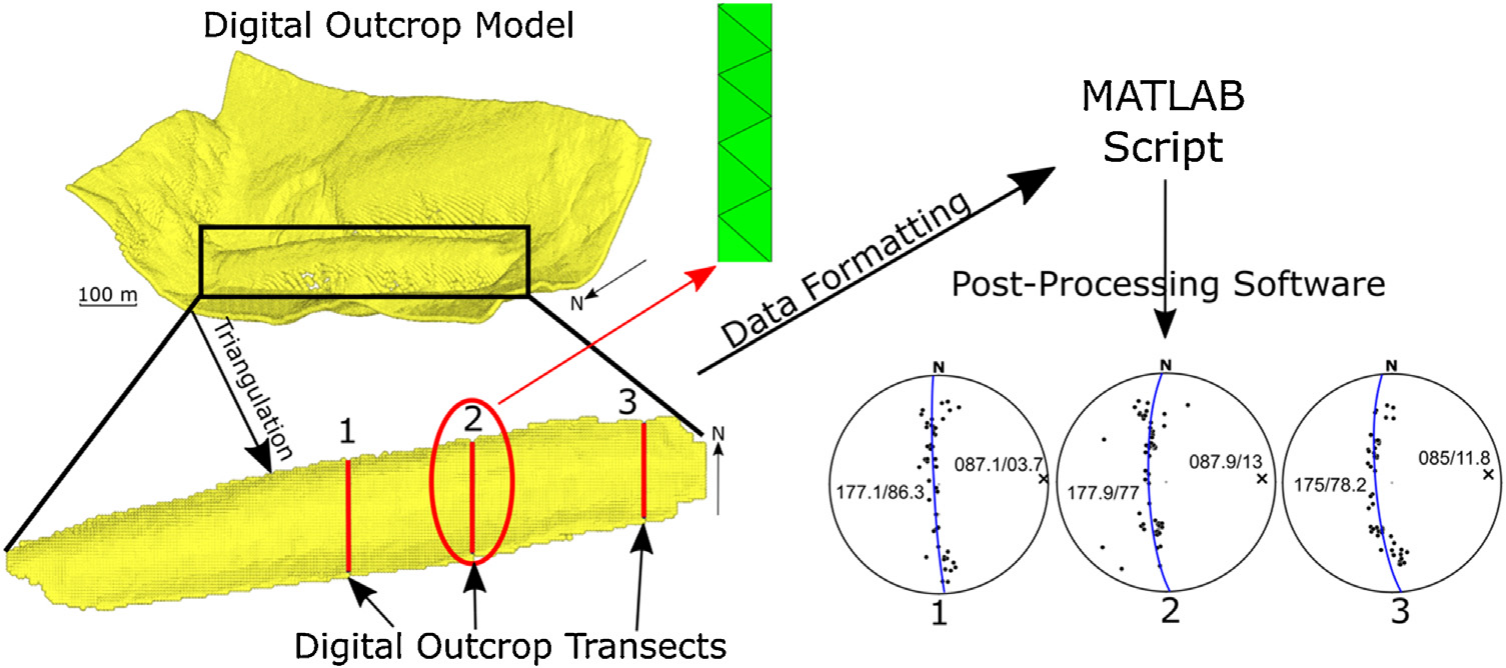
Flowchart detailing application of the workflow used to extract attitude data from a point cloud dataset of the Whaleback Pericline (Bear Valley Strip Mine, Pennsylvania). Initial dataset is a point cloud gathered by the Pennsylvania Geological Survey’s PAMAP program. Red bars across the discretized pericline surface represent virtual outcrop transects. The data representing the triangular planes (shown in green) is formatted into a spreadsheet and ran through the MATLAB script. The attitude data output can be analyzed in post-processing software, such as Stereonet 10, to further characterize results.

The described method can be applied to numerical data gathered by LiDAR to create a DEM, etc. for a more thorough analysis of fold shape, and to confirm a structural analysis performed in the field. In this example, LiDAR data gathered by the Pennsylvania Geological Survey's PAMAP program (Fig. 12) is analyzed with the script to review the cylindricity of the exposed Whaleback Anticline and the fold’s terminations (see [2] for investigational context of the Whaleback Anticline). To visualize the entire LiDAR dataset, the Cartesian coordinates were imported into Hypermesh® to be discretized into triangular surfaces. Several transects across the resulting triangular mesh were selected (i.e., mimicking virtual outcrops or cross sections of the geologic structure; see Fig. 11) for attitude analysis. Then, the coordinates of the nodes were exported and formatted (see supplemental data for example spreadsheet). Organizing the coordinates into the input spreadsheet format allows the MATLAB script to calculate the attitudes based on the triangular mesh of the transects chosen (see Fig. 2). After running the transects through the MATLAB script, extracting the strike, dip, and dip direction of each individual triangular element, the results were analyzed in Stereonet 10 [5,6]. The cylindrical best fit function of Stereonet 10 was used to visually inspect the quality of fit to the data. As shown in Fig. 12, the dataset taken from the center of the fold has a π-diagram that shows a good fit of the data to a great circle indicating a cylindrical fold shape. However, as the datasets progress towards the nose of the fold, the fit to a great circle in the π-diagrams degrades, and the data is showing a fit better suited for an ellipse. Characterization of the fold hinge line plunge using the virtually gathered π -diagrams is consistent with the hinge line plunge determined using orientation data gathered physically at the Whaleback (see [2], Fig. 5, Fig. 10). This data shows a non-consistent plunge as the measurements were taken from the center to the tip of the Whaleback and follows a generally positive trend of increasing plunge. The numerical data also reveals cones of decreasing apical angle, measured from center to tip, shown by the approximation of a small circle “tightening” when making a best fit of data. These results obtained through the structural analysis of the virtually created outcrop confirm the shape of this fold is best represented as a pericline, as shown by 3D curvature analysis rather than a conical fold (see [2] for a complete discussion).

## Declaration of Competing Interest

The authors declare that they have no known competing financial interests or personal relationships that could have appeared to influence the work reported in this paper.
